# Electronic Structure and Charge-Trapping Characteristics of the Al_2_O_3_-TiAlO-SiO_2_ Gate Stack for Nonvolatile Memory Applications

**DOI:** 10.1186/s11671-017-2040-x

**Published:** 2017-04-13

**Authors:** Wenchao Xu, Yang Zhang, Zhenjie Tang, Zhengjie Shao, Guofu Zhou, Minghui Qin, Min Zeng, Sujuan Wu, Zhang Zhang, Jinwei Gao, Xubing Lu, Junming Liu

**Affiliations:** 1 Institute for Advanced Materials, South China Academy of Advanced Optoelectronics, and Guangdong Provincial Laboratory of Quantum Engineering and Quantum Materials, South China Normal University, Guangzhou, 510006 People’s Republic of China; 2 Electronic Paper Displays Institute, South China Academy of Advanced Optoelectronics, South China Normal University, Guangzhou, 510006 People’s Republic of China; 3 College of Physics and Electronic Engineering, Anyang Normal University, Anyang, 455000 People’s Republic of China; 4 Laboratory of Solid State Microstructures and Innovation Center of Advanced Microstructures, Nanjing University, Nanjing, 210093 People’s Republic of China

**Keywords:** Charge trapping, Nonvolatile memory, High-*k* dielectrics, TiAlO

## Abstract

In this work, high-*k* composite TiAlO film has been investigated as charge-trapping material for nonvolatile memory applications. The annealing formed Al_2_O_3_-TiAlO-SiO_2_ dielectric stack demonstrates significant memory effects and excellent reliability properties. The memory device exhibits a large memory window of ~2.6 V under ±8 V sweeping voltage, and it shows only ~14% charge loss after more than 10 years’ retention, indicating excellent charge retention properties. The electronic structures of the Al_2_O_3_-TiAlO-SiO_2_ have been studied by X-ray photoelectron spectroscopy measurements, and it reveals that the quantum well and the defect traps in TiAlO film can provide a >1.8 eV deep barrier for charge confinement in the TiAlO layer. The mixing between Al_2_O_3_ and TiO_2_ can increase the defects related to the under-coordinated Ti^3+^ atoms, thereby enhancing the charge-trapping efficiency of the device. Our work implies that high-*k* TiAlO composite film is promising for applications in future nonvolatile charge-trapping memories.

## Background

Among the family of nonvolatile flash memories, charge-trapping memory (CTM) devices such as silicon-oxide-nitride-oxide-silicon (SONOS)-type memory device receive a lot of attention due to its low-operating voltage, fast program/erase (P/E) speed, good endurance, and retention characteristics over the floating-gate devices [1–3]. For these advantages, SONOS-type CTM device has been considered as a promising candidate for the next-generation nonvolatile flash memory. However, some performance and reliability issues such as low charge-trapping efficiency and poor retention characteristics still exist in SONOS-type CTM devices with the continual scaling. To overcome these disadvantages, various charge-trapping materials as well as blocking and tunneling materials were extensively investigated. Among which, high-*k* materials including thin films and their nanocrystals, for example HfO_2_, TiO_2_, and ZrO_2_, have been proposed as the charge-trapping layer in the CTM devices to achieve better storage performance and retention characteristics [4–7]. In these high-*k* dielectrics, oxygen vacancy is verified as the main origin of the defects in the film [8]. Depending on the crystal structure and method of deposition [9], TiO_2_ has a high permittivity of 80–110, which is favorable for low-voltage operation of the memory device. Another feature of TiO_2_ is that Ti has several stable oxidation states of Ti^3+^ and Ti^4+^, which leads to a well-known phenomenon with materials containing Ti-O bonds: a reduced oxide. Such a reduced oxide has many oxygen vacancies, which can act as charge-trapping centers [10]. Therefore, it is expected that TiO_2_ will be a good candidate used as the charge-trapping material, which can provide high charge-trapping ability as well as low device operation voltage. However, TiO_2_ does not have good insulating quality due to a small bandgap and low crystallization temperature [9], which is not favorable for long-term stability of the memory device. Alumina (Al_2_O_3_) has a large bandgap (~8.7 eV) and large band offsets with Si substrate [9, 11–14] and is amorphous up to high temperatures. The drawback of Al_2_O_3_ is that it only has a *k* ~ 8–10 [9], which is not favorable for the huge reduction of the operation voltage. Therefore, it is expected that the composite film of TiAlO may combine the advantages of TiO_2_ and Al_2_O_3_, which can have high charge-trapping ability, high permittivity, high thermal stability, and low leakage current at the same. Compared with the commonly studied charge-trapping materials like metal nanocrystals or monophasic high-*k* layer, the above mentioned advantages of TiAlO composite film show potentially more favorable for the high-performance operation in the future charge-trapping flash memory devices.

In this work, we first fabricated the high-*k* Al_2_O_3_/TiO_2_/Al_2_O_3_ stacking structure by electron beam evaporation. The TiAlO composite film will form by high-temperature annealing of the nominated Al_2_O_3_/TiO_2_/Al_2_O_3_ dielectric stack, and finally, we got the Al_2_O_3_/TiAlO/SiO_2_ memory device structure. The nonvolatile memory device using TiAlO composite film as charge-trapping material shows significant memory effect and excellent long-term charge stability. Although further work is still necessary to improve the overall device properties like increasing the programming speed, the TiAlO composite film is very promising for its applications in future high-performance nonvolatile memory devices.

## Methods

P-type Si (100) substrates with *ρ* = 1~10 Ω cm were first cleaned by wet-chemical solution and then dipped in a diluted HF solution (1%) to remove the surface oxide. The wafers were then immediately loaded into a vacuum chamber for deposition. The nominated Al_2_O_3_ (5 nm)-TiO_2_ (10 nm)-Al_2_O_3_ (15 nm) structure was deposited by electron beam evaporation at a substrate temperature of 300 °C. After deposition, the films were annealed at a high temperature of 900 °C in O_2_ atmosphere for 5 min by rapid thermal annealing. Dot-shaped Au top electrodes with an area of ~3.14 × 10^−4^ cm^2^ were deposited on the surface of the samples using a shadow mask by vacuum evaporation. The electrical properties of the CTM devices were characterized by an Agilent E4980A impedance analyzer and an Agilent B1500A high-precision semiconductor analyzer at room temperature. High-resolution transmission electron microscopy (HRTEM) was used to study the cross-sectional microstructures of the tri-layer dielectric stack (Tecnai G2 F20 S-Twin). The electronic structure of the memory dielectric stack was investigated by using X-ray photoelectron spectroscopy (ULVAC-PHI, PHI 5000 Versa Probe) with Al Kα X-ray source (1486.6 eV). The defect states and defect levels in our electron beam evaporation deposited TiAlO films were studied by photoluminescence (PL) (Horiba HR Revolution) measurements under 325-nm excitation wavelength.

## Results and Discussion

**Fig. 1:**
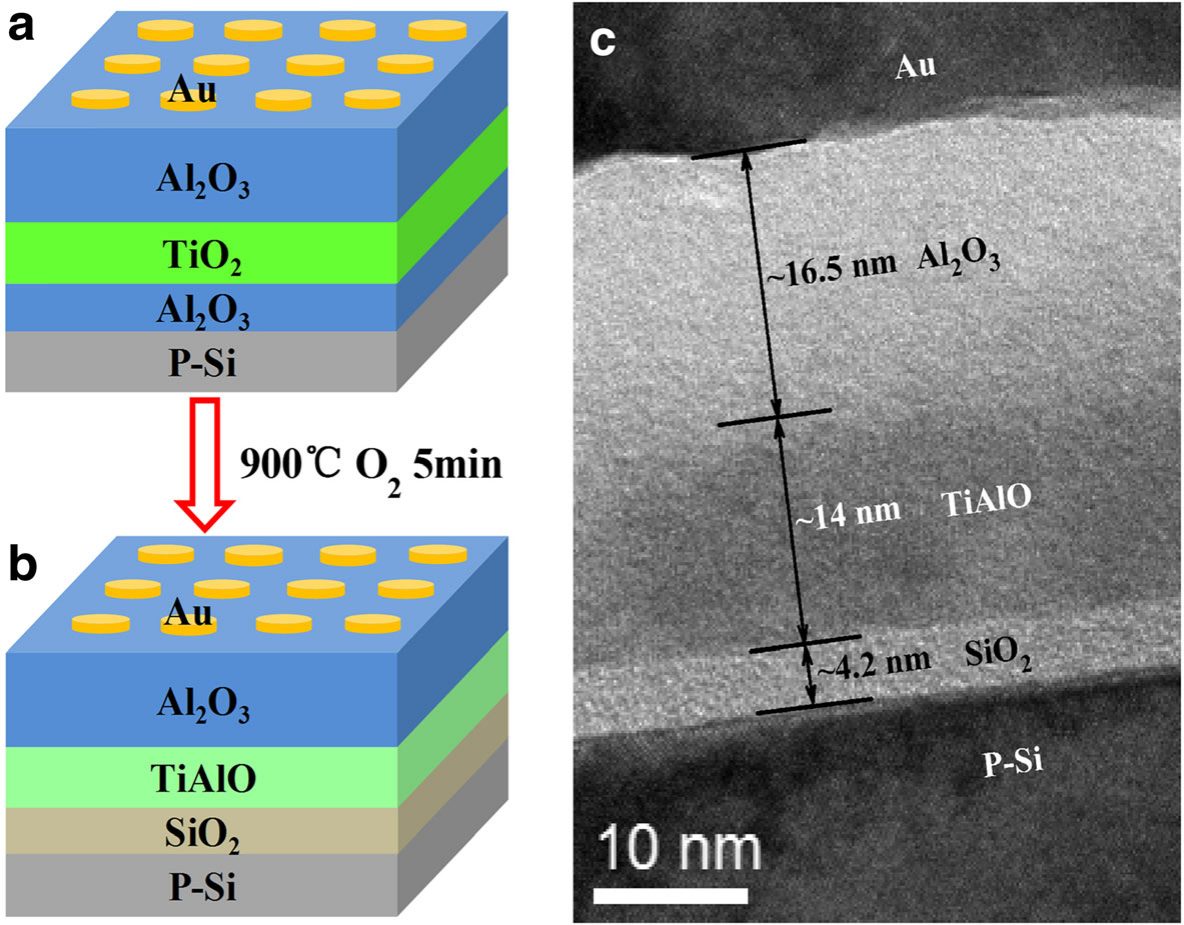
(*Color online*) **a** The schematic diagram of the nominated Al_2_O_3_/TiO_2_/Al_2_O_3_ charge-trapping memory device structure. **b** The schematic diagram of the charge-trapping memory device structure after 900 °C annealing. **c** The HRTEM cross-sectional image of the actual charge-trapping memory device used in our work

Figure 1a, b shows the schematic diagrams of the tri-layer dielectric stack before and after high-temperature annealing. Before annealing, a nominated Al_2_O_3_ (5 nm)-TiO_2_ (10 nm)-Al_2_O_3_ (15 nm) charge-trapping memory structure was deposited by electron beam evaporation. After 900 °C annealing in oxygen for 5 min, the memory structure will change to a SiO_2_ (~4.2 nm)-TiAlO (~14 nm)-Al_2_O_3_ (~16.5 nm) structure, as shown in Fig. 1b. Figure 1c shows the cross-sectional HRTEM image of the actual memory structure after annealing. One amorphous SiO_2_ layer of around 4.2 nm can be clearly observed. Under high-temperature annealing, the formation of interfacial SiO_2_ layer has been often observed [7]. Noticeably, the 5-nm-thick tunneling layer of Al_2_O_3_ disappeared, which was ascribed to the mixture of the tunneling Al_2_O_3_ layer and TiO_2_. It is most probably that the mixture in the interface between the blocking Al_2_O_3_ layer and TiO_2_ also occurred. The thickness of the composite TiAlO layer and Al_2_O_3_ blocking layer are ~14 and 16.5 nm, respectively. The TiO_2_/Al_2_O_3_ mixture under high temperature has been reported by other researchers [15–17]. For example, in work by Mikhelashvili et al. [16] after annealing at 950 °C, the Al_2_O_3_-TiO_2_ nanolaminates transformed into a TiAlO layer with a nearly uniform distribution of Al and Ti oxides across the structure. Actually, this behavior is initiated already at 550 and at 750 °C in their work. Further, XPS study in our work also confirmed this mixture between TiO_2_ and Al_2_O_3_. Therefore, the actual high-temperature annealed memory dielectric stack in this work is Al_2_O_3_-TiAlO-SiO_2_.

**Fig. 2:**
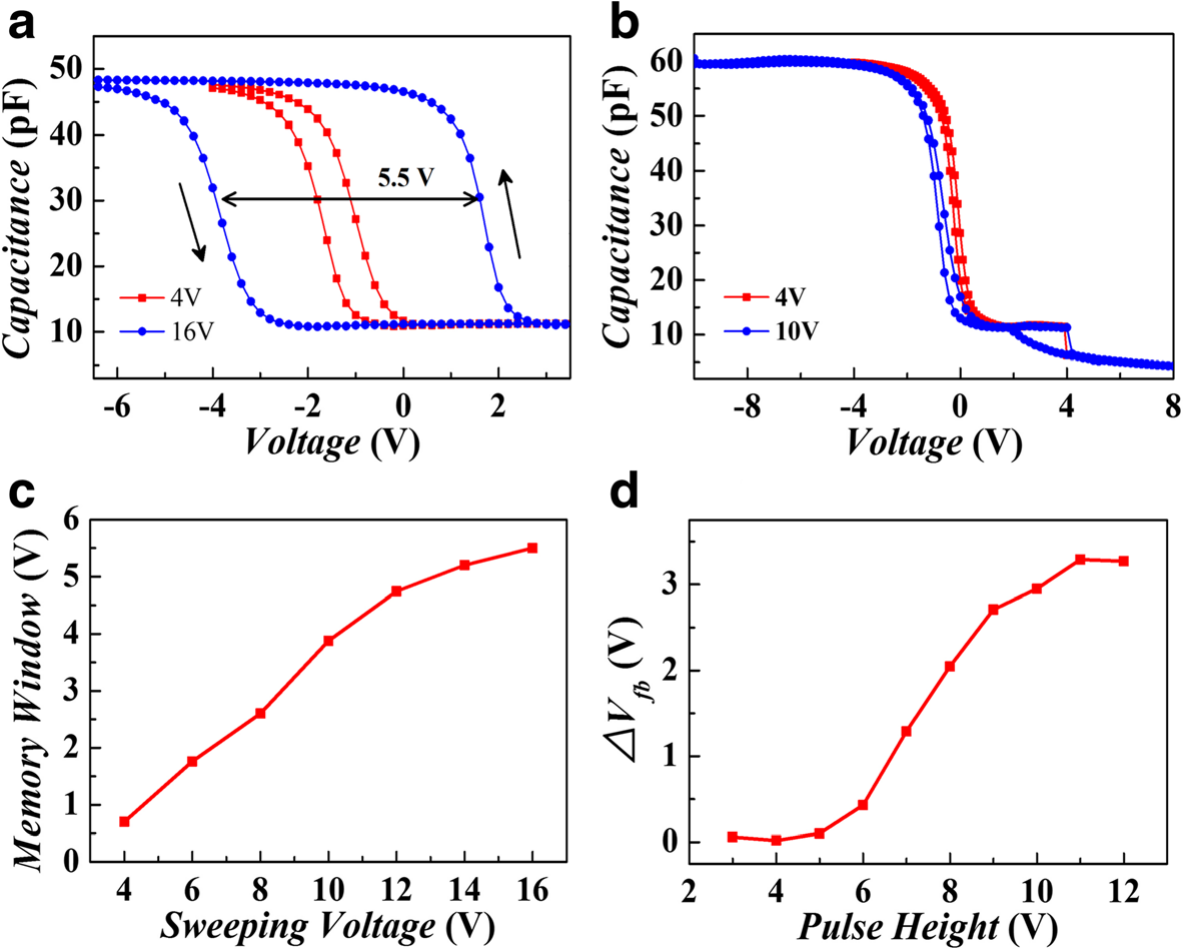
(*Color online*) Typical high-frequency (1 MHz) CV characteristics of the Au-Al_2_O_3_-TiAlO-SiO_2_-Si device (**a**) and Au-Al_2_O_3_-SiO_2_-Si device (**b**). **c** Memory window width dependence of the Au-Al_2_O_3_-TiAlO-SiO_2_-Si device under different sweeping voltages. **d** Dependence of the memory window width on the pulsed writing voltage height, the program/erase pulse width is fixed to be 1 s

Figure 2a shows the typical high-frequency (1 MHz) capacitance-voltage (CV) characteristics of the Au-Al_2_O_3_-TiAlO-SiO_2_-Si devices under ±4 and ±16 V. The anticlockwise direction of the CV hysteresis demonstrates the typical hysteresis loop directions of charge trapping. To confirm that memory effect is really from TiAlO composite film, we also fabricated a single layer of 20-nm-thick Al_2_O_3_ using electron beam evaporation, and it also receives an annealing at 900 °C for 5 min in O_2_. Figure 2b shows the typical high-frequency (1 MHz) CV characteristics of the Au-Al_2_O_3_-SiO_2_-Si devices under ±4 and ±10 V. It was clear that no obvious memory effect can be observed for the diode device based on single-layer Al_2_O_3_ film. Only a hysteresis loop around 0.21 V was observed for both scanning voltages of ±4 and ±10 V. The results shown in Fig. 2b proved that the significant memory effect of Al_2_O_3_-TiAlO-SiO_2_ really comes from the charge trapping of TiAlO layer. For both of the Al_2_O_3_-TiAlO-SiO_2_ and Al_2_O_3_-SiO_2_ gate stack structure, a small hysteresis loop can be observed under ±4 V scanning voltages, which is most probably due to charge trapping/de-trapping in the shallow traps in the Si/SiO_2_ interface.

Figure 2c shows the dependence of the memory window on the different sweeping voltages. The memory window increases with the increase of the sweeping voltages, and it tends to be saturated around 16 V sweeping voltages. A large memory window of 5.5 V under ±16 V sweeping voltage indicates significant charge-trapping effect in the TiAlO charge-trapping layer. Even for small scanning gate voltages of ±6 and ±8 V, comparatively large memory windows of 1.8 and 2.6 V can be observed. The P/E characteristics of the memory device were also investigated by measuring the flat voltage shift (Δ*V*_fb_) induced by a pulsed P/E voltage. Pulses with different pulse height (ranging from 3 to 12 V) and same pulse width (1 s) were applied onto the diode. The change of the flat-band voltage (*V*_fb_) shift with the pulse height was shown in Fig. 2d. The *V*_fb_ calculation method is the same to that we used in previous work [18]. Memory effect can be clearly observed even under a small P/E voltage of 6 V. Large Δ*V*_fb_ value of ~3.0 V can be observed under 10 V P/E voltages, demonstrating its low-voltage operations compared with the conventional flash memories. It should be mentioned that the operation speed of the present device is still low. One of the reasons may be that the films were deposited by electron beam evaporation, which is likely to have lots of defects that could limit the P/E performance of the memory device. Charge-trapping memory devices fabricated by atomic layer deposition (ALD) exhibits excellent electrical performances [12, 19]. In the future, if we can use high-quality film growth method such as ALD to fabricate the dielectric stack, the electrical performance of the present TiAlO charge-trapping memory device may have a lot of space to be further improved.

**Fig. 3:**
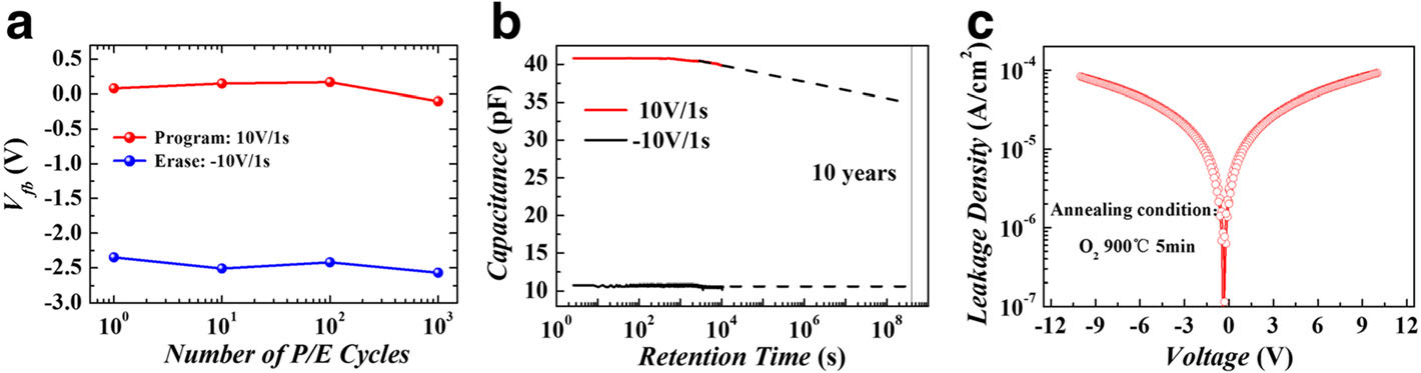
(*Color online*) The endurance (**a**), retention (**b**), and leakage current-voltage (**c**) characteristics for the Au-Al_2_O_3_-TiAlO-SiO_2_-Si device

Figure 3a illustrates the endurance property of the memory device at room temperature. After 10^3^ P/E processes, the memory window hardly changes, indicating good endurance characteristics. As shown in Fig. 3b, the memory device also exhibits excellent retention characteristics. The high and low capacitance demonstrates to be very stable under −1 V read voltage. After 10^4^ s’ retention, only 2% of the high capacitance was lost. Even extrapolated for 10 years, only 14% loss of the high capacitance was observed. In the low capacitance remains nearly unchanged during all the measurement time. Figure 3c shows the leakage current characteristics of the memory device. The leakage current under −10 V bias voltage is determined to be 8.0 × 10^−5^ A/cm^2^. For the P/E process by using pulse with 10 V in height and 1 s in width, the tunneling current responsible for charge-trapping memory effect can be estimated to be 4.78 × 10^−7^ A/cm^2^, which is much smaller than that of the leakage current. This might be the main reason of the long program and erase time. The reason for this big difference is not very clear now, which is assumed to be closely related to the film quality deposited by electron beam evaporation. If high-quality film growth method such as atomic layer deposition is used to deposit TiAlO charge-trapping material and Al_2_O_3_ blocking oxide, the P/E speed of the memory device can be expected to have a big improvement.

**Fig. 4:**
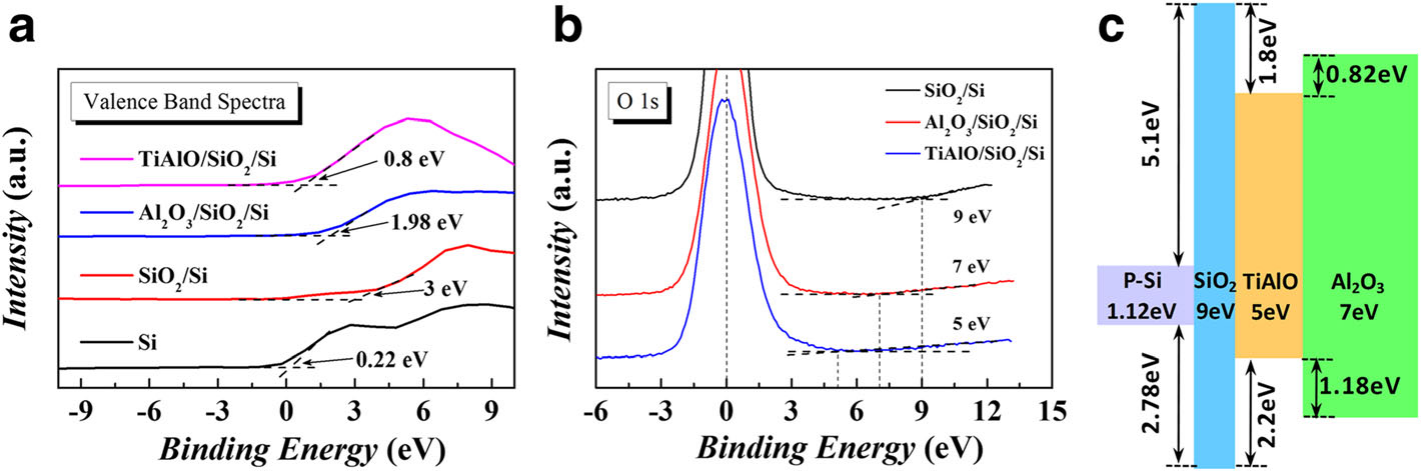
(*Color online*) **a** Valance band spectra of P-Si substrate, SiO_2_/Si, TiAlO/SiO_2_/Si, and Al_2_O_3_/SiO_2_/Si structure. **b** O 1 s electron energy loss spectra of SiO_2_/Si, TiAlO/SiO_2_/Si, and Al_2_O_3_/SiO_2_/Si structure. **c** A schematic diagram of the band alignments of the Al_2_O_3_-TiAlO-SiO_2_-Silicon structure

Considering the charge-trapping memory devices, electronic structure of the tri-layer dielectric stack (Al_2_O_3_-TiAlO-SiO_2_) should be an important mechanism to affect the memory characteristics. The band alignment of the Al_2_O_3_-TiAlO-SiO_2_ dielectric stack was investigated by valence band and energy loss spectra by using XPS, as shown in Fig. 4. The XPS measurement results of valance band spectra are shown in Fig. 4a. By using a linear extrapolation method [20], the valence band maximums (VBM) of P-Si substrate, SiO_2_/Si, TiAlO/SiO_2_/Si, and Al_2_O_3_/SiO_2_/Si structure were determined to be 0.22 eV $\left({E}_{VBM}^{Si}\right)$, 3 eV $\left({E}_{VBM}^{Si{ O}_2}\right)$, 0.8 eV $\left({E}_{VBM}^{TiAlO}\right)$, and 1.98 eV $\left({E}_{VBM}^{A{ l}_2{O}_3}\right)$, respectively. Therefore, the valance band offsets of SiO_2_/Si $\left(\varDelta {E}_V^{Si{ O}_2/ Si}\right)$, TiAlO/SiO_2_ $\left(\varDelta {E}_V^{TiAlO/ Si{O}_2}\right)$, and Al_2_O_3_/TiAlO $\left(\varDelta {E}_V^{A{ l}_2{O}_3/ TiAlO}\right)$ were calculated as 2.78, −2.2, 1.18 eV, respectively, by using the following equations:

1 $$ \varDelta {E}_V^{{\mathrm{Si}\mathrm{O}}_2/\mathrm{Si}}={E}_{\mathrm{VBM}}^{{\mathrm{Si}\mathrm{O}}_2}-{E}_{\mathrm{VBM}}^{\mathrm{Si}}, $$

2 $$ \varDelta {E}_V^{{\mathrm{TiAlO}/\mathrm{SiO}}_2}={E}_{\mathrm{VBM}}^{\mathrm{TiAlO}}-{E}_{\mathrm{VBM}}^{{\mathrm{SiO}}_2}, $$

3 $$ \varDelta {E}_V^{Al_2{O}_3/ T i A l O}={E}_{V BM}^{Al_2{O}_3}-{E}_{V BM}^{TiAlO} , $$

The bandgaps of SiO_2_, Al_2_O_3_, and TiAlO were determined by the onset of O 1 s electron energy loss spectra [20], as shown in Fig. 4b. For SiO_2_, Al_2_O_3_, and TiAlO, the bandgaps were determined as 9 eV $\left({E}_g^{Si{ O}_2}\right)$, 7 eV $\left({E}_g^{A{ l}_2{O}_3}\right)$, and 5 eV $\left({E}_g^{TiAlO}\right)$, respectively. The conduction band offset (CBO) of SiO_2_/Si $\left(\varDelta {E}_C^{Si{ O}_2/ Si}\right)$, TiAlO/SiO_2_ $\left(\varDelta {E}_C^{TiAlo/ Si{O}_2}\right)$, and Al_2_O_3_/TiAlO $\left(\varDelta {E}_C^{A{ l}_2{O}_3/ TiAlO}\right)$ could be deduced by using the following formulas:

4 $$ \varDelta {E}_C^{{\mathrm{Si}\mathrm{O}}_2/\mathrm{Si}}={E}_g^{{\mathrm{Si}\mathrm{O}}_2}-{E}_g^{\mathrm{Si}}-\varDelta {E}_V^{{\mathrm{Si}\mathrm{O}}_2/\mathrm{Si}}, $$

5 $$ \varDelta {E}_C^{{\mathrm{TiAlO}/\mathrm{SiO}}_2}={E}_g^{\mathrm{TiAlO}}-{E}_g^{{\mathrm{SiO}}_2}-\varDelta {E}_V^{{\mathrm{TiAlO}/\mathrm{SiO}}_2}, $$

6 $$ \varDelta {E}_C^{{\mathrm{Al}}_2{\mathrm{O}}_3/\mathrm{TiAlO}}={E}_g^{{\mathrm{Al}}_2{\mathrm{O}}_3}-{E}_g^{\mathrm{TiAlO}}-\varDelta {E}_V^{{\mathrm{Al}}_2{\mathrm{O}}_3/\mathrm{TiAlO}}, $$

where ${E}_g^{{\mathrm{SiO}}_2}$ is 1.12 eV. The deduced values of CBO were 5.1, −1.8, and 0.82 eV for SiO_2_/Si, TiAlO/SiO_2_, and Al_2_O_3_/TiAlO, respectively. Based on the above data, the complete band alignments of our memory devices can be established, as schematically shown in Fig. 4c. After programming, the charges will be trapped in the SiO_2_-TiAlO-Al_2_O_3_ quantum well and the defect states of TiAlO film. Since $\varDelta {E}_C^{{\mathrm{TiAlO}/\mathrm{SiO}}_2}$ is 1.8 eV, the electrons need to overcome at least a barrier of 1.8 eV to tunnel from the TiAlO charge-trapping layer back to the Si substrate. For the charges trapped in the defect states, for example oxygen vacancy, in the forbidden band of TiAlO, the barrier should be higher than 1.8 eV. This deep barrier for charge trapping is assumed to be the most important reason for the excellent retention properties of the present memory device.

**Fig. 5:**
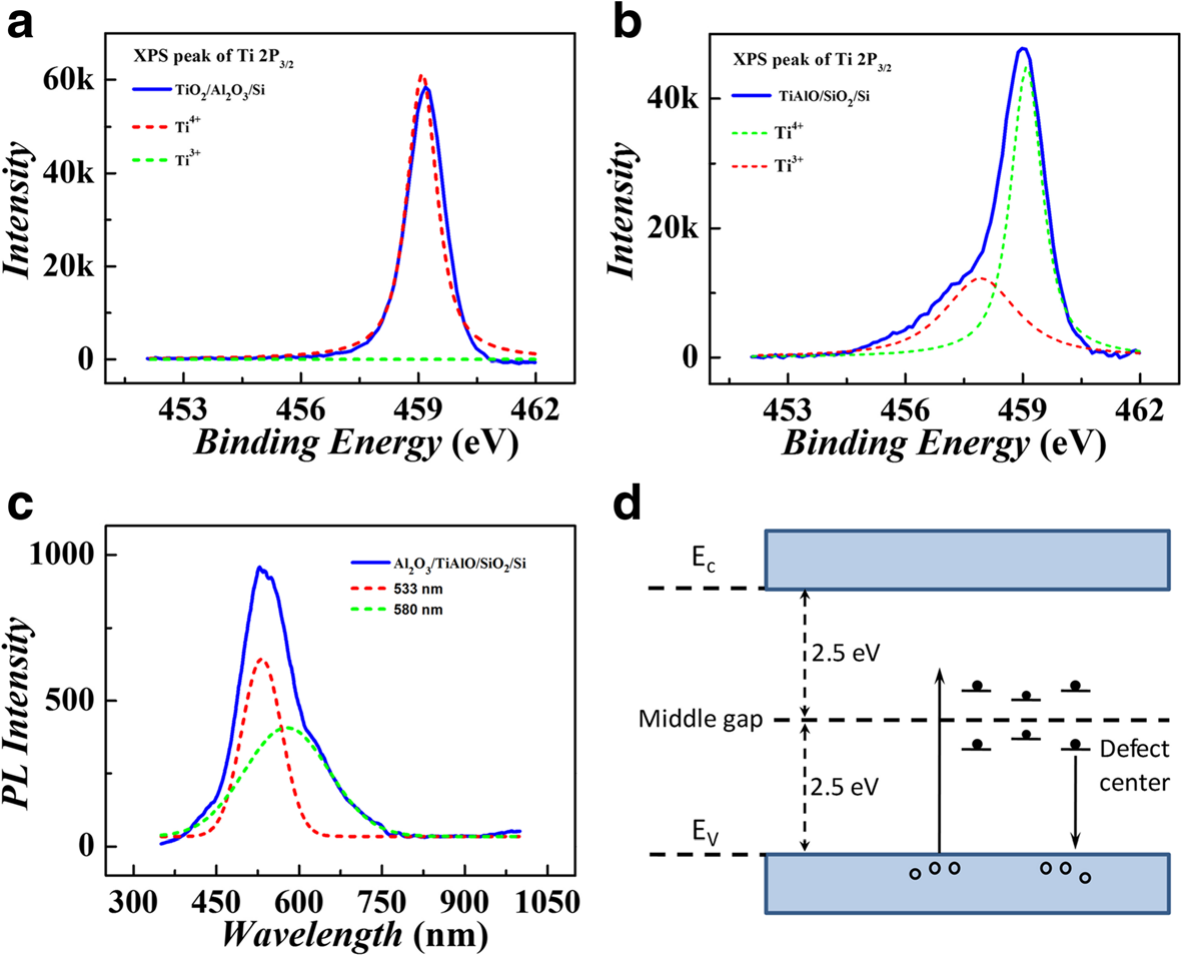
(*Color online*) XPS spectra of Ti 2p_3/2_ states from the TiO_2_/Al_2_O_3_/Si (**a**) and TiAlO/SiO_2_/Si (**b**) structure. **c** Room-temperature PL spectra from Al_2_O_3_/TiAlO/SiO_2_ structure. **d** A schematic diagram of the defect levels and PL processes in TiAlO film

To investigate the charge-trapping mechanisms in our device, electron beam evaporation deposited TiAlO films were further studied by XPS measurement. Figure 5a, b shows the Ti 2p_3/2_ XPS spectrum of the TiO_2_/Al_2_O_3_/Si structures with and without 900 °C annealing, respectively. By fitting the spectrum with a Gaussian function [10, 21], the Ti 2p_3/2_ XPS spectrum of annealed structure (TiAlO/SiO_2_/Si) can be resolved into two individual peaks, as shown by dotted lines in Fig. 5b. These two peaks could be ascribed to the contributions of Ti^3+^ 2p_3/2_ and Ti^4+^ 2p_3/2_. Jiang et al. [10] had reported that in (TiO_2_)_0.8_(Al_2_O_3_)_0.1_ film part of Ti^4+^ ions were transformed into Ti^3+^ ions due to the diffusion between TiO_2_ and Al_2_O_3_. Therefore, in the TiAlO/SiO_2_/Si structure, a small amount of trivalent Ti^3+^ ions formed because of the mixing of Al_2_O_3_ and TiO_2_. Jin et al. [21] had found that the electron traps associated with the under-coordinated Ti^3+^ atoms can capture electrons. Thus, the TiAlO mixture formed by annealing can increase the defect density and may enhance the efficiency of the electron trapping. Different from Fig. 5b, the XPS spectra of the un-annealed TiO_2_/Al_2_O_3_/Si structure shown in Fig. 5a can be well fitted by Ti^4+^ 2p_3/2_ peak, and Ti^3+^ 2p_3/2_ signal cannot be observed. The present Ti 2p_3/2_ XPS results proved again the mixture of alumina and titanium oxide after high-temperature annealing, and the mixed TiAlO composite film is expected to improve the efficiency of charge trapping.

The defect level in TiAlO composite film was characterized by the PL spectra, as shown in Fig. 5c. Through fitting the spectrum with a Gaussian function, the PL spectra can be divided into two separate peaks. The red dotted fitting line shows a PL peak at 533 nm, and the green dotted fitting line shows a PL peak at 580 nm. The PL peak at ~580 nm is believed to have a strong correlation with the defects associated with under-coordinated Ti^3+^ ions, and the PL peak at ~533 nm is related to the oxygen vacancies [21–23]. In addition to the defects caused by the under-coordinated Ti^3+^ ions, the charge-trapping effects in TiAlO composite film may be also partly from oxygen vacancy-related defects. Figure 5d shows a schematic diagram of the PL process in our devices. Since the bandgap of TiAlO was determined to be 5 eV by XPS, the PL emission should not come from the band to band emission. The electrons may be excited to the defect levels in the bandgap of TiAlO, whose center position is around 2.3 eV (533 nm) above the top of valence band. The excited electrons in defect levels then recombined with the holes in the valence band. Based on the PL results, we believe that the center of the defect levels in TiAlO film is located close to the middle gap of TiAlO, which are deep traps for charge trapping in our memory devices. The deep trap levels in TiAlO composite film are expected to be one of the critical reasons for the excellent charge retention in the present memory devices.

**Fig. 6:**
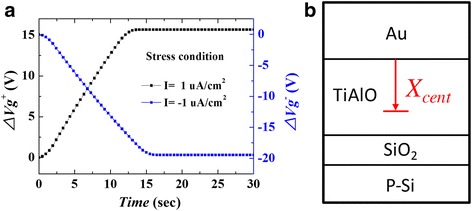
(*Color online*) **a** Charge-trapping characteristics of the Au/TiAlO/SiO_2_/Si structure under constant current stress of 1 μA/cm^2^. **b** A schematic diagram of the charge trap centroid (*X*_cent_) of the TiAlO film

The charge trap centroid of TiAlO was evaluated using the constant current stress (CCS) method [24]. Figure 6a shows the charge-trapping characteristics under a constant current of 1 μA/cm^2^. The voltage drop at TiAlO/SiO_2_-stacked layer was measured as the gate voltage shift varied with increasing stress time. The shift in gate voltage is attributed to the charge trapping in TiAlO layer. The voltage shift was observed from the TiAlO/SiO_2_-stacked layer, and the amount of voltage shift increased with stress time. Accordingly, we can conclude that the TiAlO layer has good charge-trapping characteristics. The charge trap centroid (*X*_cent_) was extracted by using the CCS measurement method [24],

7 $$ {X}_{\mathrm{cent}}=\frac{t_{\mathrm{stack}}}{\left[1-\left(\varDelta {V}_g^{-}/\varDelta {V}_g^{+}\right)\right]}, $$

where *X*_cent_ is measured from the metal gate/oxide interface and $\varDelta {V}_g^{-}$ and $\varDelta {V}_g^{+}$ are the negative and positive gate voltage shifts, respectively. The calculated *X*_cent_ is 6.7 nm, which is nearly close to the middle of TiAlO charge-trapping layer. Figure 6b shows the schematic diagram of *X*_cent_ position of the Au/TiAlO/SiO_2_/Si structure.

## Conclusions

In this paper, the nominated Al_2_O_3_-TiO_2_-Al_2_O_3_ tri-layer charge-trapping memory structure was fabricated by electron beam evaporation, and the tri-layer dielectric stack changed to Al_2_O_3_-TiAlO-SiO_2_ structure after annealing at 900 °C. The annealing formed memory devices with high-*k* TiAlO charge-trapping layer exhibit significant memory effects and excellent reliability properties. The electronic structures of the tri-layer dielectric stack (Al_2_O_3_-TiAlO-SiO_2_) were investigated by valence band and energy loss spectra measurements of XPS. The deep barrier height for charge confinement in TiAlO layer and good insulating properties of the gate dielectric were believed to be the reasons for the excellent retention and endurance properties of the memory device. The mixing between Al_2_O_3_ and TiO_2_ can increase the defects related to the under-coordinated Ti^3+^ atoms, thereby enhancing the charge-trapping efficiency of the device. The defect level center of the high-*k* TiAlO is determined to be located at the middle gap of TiAlO film by PL measurement. Our results imply that the high-temperature annealing formed high-*k* TiAlO composite film is promising for applications in the future nonvolatile memories.

## Abbreviations

ALD: Atomic layer deposition
CBO: Conduction band offset
CCS: Constant current stress
CTM: Charge-trapping memory
CV: Capacitance-voltage
P/E: Program/erase
PL: Photoluminescence
VBM: Valence band maximums
*X*_cent_: Charge trap centroid
XPS: X-ray photoelectron spectroscopy
